# Editorial for the Special Issue “Phytotoxicity of Heavy Metals in Contaminated Soils”

**DOI:** 10.3390/toxics11060536

**Published:** 2023-06-16

**Authors:** Ana Romero-Freire, Manuel Sierra-Aragón, Hao Qiu, Erkai He

**Affiliations:** 1 Department Soil Science and Agricultural Chemistry, University of Granada, 18071 Granada, Spain; miserra@ugr.es; 2 School of Environmental Science and Engineering, Shanghai Jiao Tong University, Shanghai 200240, China; haoqiu@sjtu.edu.cn; 3 School of Geographic Sciences, East China Normal University, Shanghai 200241, China; ekhe@geo.ecnu.edu.cn

Soil is an essential natural resource because of the ecosystem services it carries out in the terrestrial ecosystem: the provision of food, fibre and fuel; habitats for organisms; nutrient cycling; climate regulation and carbon sequestration; water purification and soil contaminant reductions; and others. However, human activities are the main cause of soil degradation, leading to soil loss through erosion, fertility decline or even generating toxicity problems that pose a risk not only to plants and organisms but also to human health.

Soil contamination is a major environmental problem worldwide, which is why control and remediation measures for contaminated soils should be carried out to prevent the spread of contamination and toxicity derived from the accumulation of pollutants in the soil. Potentially toxic elements, which include metals and metalloids, are of particular relevance because of the large number of contaminated sites worldwide, the toxicity risks they pose to plants and organisms and the persistence of these toxins in soils.

Heavy metals can cause a variety of phytotoxic effects, such as growth inhibition, oxidative stress due to the presence of reactive oxygen species, instability of cell membranes, alteration in enzyme activity, reduction in chlorophyll content and leaf water potential and/or alteration in stomatic function. Moreover, the phytotoxicity produced by heavy metals in contaminated soils may result in reduced plant species biodiversity compared to uncontaminated soils. Singh and co-authors [1] report how Pb contamination of soils due to coal fly ash dumping from a thermal power plant reduced the plant species count, abundance, density and richness compared to an uncontaminated area nearby. In addition, morphological differences, such as seed morphology, plant height and stomata size, were observed in the same plant species in both Pb-contaminated and non-polluted areas.

Heavy metals can also cause phytotoxic effects at the genetic level. At high concentrations, heavy metals act as catalysts in the oxidative degradation of biological macromolecules, leading to DNA damage by oxidative stress. Bölükbaşı and Karakaş [2] exposed *Carthamus tinctorius* L. (Safflower) seeds to different concentrations of Cu heavy metal solution and reported that high doses of Cu have a genotoxic effect on safflower plants. The presence of a substantial amount of polymorphism in the safflower plants exposed to Cu heavy metal stress was observed, as well as altered DNA methylation patterns, a crucial defence mechanism utilized by the safflower plant. The hazard from soils contaminated by heavy metals and other potentially toxic elements depends on the risk of their toxicity, which depends on the bioavailability of the pollutant in the soil. Therefore, in ecosystems with soils contaminated by heavy metals, it is not only necessary to identify the pollutants, but also to determine their concentration, fractionation and geographical distribution, analyse their ecotoxicity risks and finally develop recovery plans for the contaminated soils.

The presence of heavy metals in soils is related to different human activities, such as metallic mining, industrial activities and thermal power plants, among others. Once contaminated areas re delimited, it is necessary to know the distribution of metals in the area, as they can condition the distribution of vegetation in terms of cover, plant structures and diversity. In this sense, González-Morales and co-authors [3] analyse the distribution pattern of metals (Pb, Zn, Tl and Cr) in an abandoned sphalerite mining area using Geographic Information Systems, allowing the geographical identification of the contamination source areas and their influence on the vegetation cover, as well as identifying metal-accumulating species such as *Q. rotundifolia* Lam, *S. holochoenus* (L.) Soják, *Carlina ssp*. L. and *R. sphaerocarpa* (L.) Boiss. Among these species, *R. sphaerocarpa* was the most suitable for the application of revegetation measures in the area as part of the restoration plan for the contaminated area due to its good adaptation to the environment and its capacity to bioaccumulate metals.

The fractionation of heavy metals in the soil is directly influenced by soil properties, conditioning their solubility, mobility and bioavailability in the soil and ultimately their toxicity. Kharazian and co-authors [4] used the European Community Bureau of Reference (BCR) three-step sequential extraction method to identify the fractionation of heavy metals (Zn, Cd and Pb) in soils with a *P. halepensis* vegetation cover from an old mine located in the Metalliferous Ring of the Sulcis–Iglesiente mining district (Sardinia, Italy). The relationship between soil properties, metal fractionation and metal concentration in the plant allowed for the evaluation of the bioavailability of metals, showing that Cd was more available in pine trees while Zn and Pb, which are mainly associated with the BCR residual fraction, showed a low availability and bioaccumulation in pine trees.

The bioavailability and plant uptake capacity of heavy metals also depends on the concentration of nutrients in the soil solution. Wang and co-authors [5] observed how Cd uptake by *Bidens pilosa* L. changes in a 10 μM Cd^2+^ solution by varying the concentration of nutrients (Ca^2+^, Mg^2+^, Fe^2+^, SO_4_^2−^, Na^+^ and K^+^). High nutrient concentrations showed an antagonistic effect on root uptake of Cd^2+^, while Fe^2+^, at low concentrations, showed a synergistic effect on Cd^2+^ uptake, forming an oxide membrane on the root surface to promote Cd uptake by *B. pilosa*.

Once the contaminated area has been identified, it is necessary to develop soil remediation actions to reduce the toxicity risks. Paniagua-López and co-authors [6] analysed a method of reducing toxicity through the addition of amendments (gypsum, marble, vermicompost) to soils contaminated by heavy metals and associated elements (As, Cd, Cu, Pb and Zn) affected by a mining spill of pyritic sludge. The treatment based on the addition of marble sludge together with vermicompost demonstrated the best results according to different toxicity bioassays (reduction in root elongation of lettuce seeds, root elongation of barley plants and inhibition of algae growth).

Heavy metal phytotoxicity research on contaminated soils using different approaches, including determining the soil mechanisms that control the bioavailability of heavy metals in the soil, the absorption capacity of these metals by the plant and their translocation capacity and defence mechanisms, will be fundamental to developing a reliable toxicity risk assessment and enabling a rational development of remediation programmes for heavy-metal-contaminated soils.
